# Enhancing implementation of a standardized initial assessment for demand management in outpatient emergency care in Germany: a quantitative process evaluation

**DOI:** 10.1186/s12911-021-01685-6

**Published:** 2021-11-16

**Authors:** Amanda Breckner, Catharina Roth, Joachim Szecsenyi, Michel Wensing

**Affiliations:** Department of General Practice and Health Services Research. Marsilius Arcades, Heidelberg University Hospital, West Tower, Im Neuenheimer Feld 130.3, 69120 Heidelberg, Germany

**Keywords:** Emergency medical services, Outpatient emergency care service, Software, Point-of-care systems, Germany

## Abstract

**Background:**

Inadequate assessment of the severity and urgency of health problems is one of the factors contributing to unnecessary emergency department visits. A software-based instrument for standardized initial assessment (SmED) aims to support healthcare professionals and steer patients to the appropriate source of care. The aim of this study was to evaluate the implementation process of SmED based on the point of view of users in order to facilitate sustainable implementation.

**Methods:**

A quantitative process evaluation on the basis of a paper-based questionnaire was carried out alongside the implementation of SmED in 26 outpatient emergency care services within 11 federal states in Germany. Healthcare professionals who worked with SmED either at the joint contact points of the outpatient emergency care service and the emergency departments of hospitals ("Joint Counter", German “Gemeinsamer Tresen”) or at the initial telephone contact points of the outpatient emergency care service (116117) were invited to participate in the survey.

**Results:**

200 users of SmED completed the questionnaire comprising the five scales: Intervention effectiveness/efficacy, Interprofessional context/occupational Interest, Individual Context, Organisational Framework Conditions, and Medical Context. Several individual characteristics were related to the implementation process of SmED. Female and younger healthcare professionals and participants with less than five years of professional experience tended to evaluate the implementation process as more positive. Factors related to the Individual Context and to the Medical Context were associated with the reported use of SmED (*p* = 0.004 and 0.041, respectively).

**Conclusion:**

The involvement of healthcare professionals, particularly more experienced professionals, in the implementation of SmED may help to facilitate sustainable implementation. In addition, training of potential user prior and during the implementation process and the adaption of Organisational Context factors are crucial.

*Trial registration* The study was registered at the German Clinical Trials Register prior to the start of the study (DRKS00017014).

**Supplementary Information:**

The online version contains supplementary material available at 10.1186/s12911-021-01685-6.

## Background

The increasing utilisation of emergency departments (EDs) is a common challenge in western countries [1–4]. One factor which contributes to the overuse of emergency resources are unnecessary ED visits. These visits are defined as unnecessary because ED resources are used for health conditions, which do not require immediate medical treatment and could be treated elsewhere e.g. during office-hours in primary medical care [5]. An estimate of 20 to 40% of all visits to EDs are unnecessary [3, 5]. Factors which are related to unnecessary ED visits include lower patient age, lower education, and absence of family support [3]. Moreover, the accuracy of the assessment of severity and urgency of the presented health issue determines the number of unnecessary ED visits [3, 4, 6, 7]. Structured assessment of the urgency of demand at the point of entry to emergency care may help to reduce the burden on EDs. Therefore, a computer-based software (SmED) was developed and implemented in two out-of-hours service settings (Joint Counter (German: Gemeinsamer Tresen) and the initial telephone contact point) in Germany to support healthcare professionals to steer patients into the right source of care and thus reduce unnecessary ED visits since March 2019 [8, 9].

Implementing complex interventions, like a computer-based decision support, is challenging. Complex interventions often fail to be implemented comprehensively and sustainably in healthcare practice [10]. Different factors act as barriers or facilitators of the implementation process including e.g. interprofessional or individual context factors or organisational framework conditions [11, 12]. It is crucial to identify and explore these factors in order to enhance sustainable implementation [10]. Furthermore, different user characteristics could predict the implementation process of SmED. Therefore, it is crucial to involve user experiences and perceptions in the process evaluation to facilitate sustainable implementation and to improve quality of healthcare services [13]. In addition, to improve healthcare and to enhance the implementation process it is important to understand the association between specific user characteristics and factors that predict the uptake in healthcare practice. Moreover, user acceptance has been shown to be the central dimension of the support of clinical decision support system [21].

Research has shown that technology innovations in healthcare often meet a variety of challenges such as the resistance from healthcare professionals, perceived impracticability by potential users, the overall complexity of the innovation, different characteristics of potential users, or simply that the innovation is not adapted to the setting [13, 14]. This in turn has an impact on the success of the implementation process and the sustainability of the innovation. A good understanding of the user perspective on technological innovations, such as computerized decision support systems like SmED, is therefore crucial for effective implementation. Furthermore, within the setting of out-of-hours care in other countries it has been shown that telephone triage handled by nurses or other healthcare professionals make a crucial contribution to patient management [19, 20]. Nevertheless, user perspectives and characteristics of users have not been on the forefront of research in this setting, as it is that patients or protocols of telephones calls were predominantly analysed.

The aim of this study was to evaluate the implementation process of SmED from the point of view of the software users in order to facilitate sustainable implementation. In addition, we explored the associations between the views on the uptake of SmED, on reported use of SmED, and individual characteristics. Due to structural differences we furthermore compared the two out-of-hours service settings.

## Methods

### Study design and study setting

The research presented in this manuscript is part of the DEMAND project [9], which aimed to improve medical care of patients who present an urgent need for emergency treatment and/or medical advice on the basis of a more efficient use of emergency care resources. In March 2019 a software-based instrument for standardized initial assessment (SmED) was implemented by the Associations of Statutory Health Insurance Physicians to support healthcare professionals (e.g. nurses, physician assistants, paramedics) in emergency care. SmED is currently used at the Joint Counter (German: Gemeinsamer Tresen), a cooperative arrangement between the outpatient emergency care service and the EDs of the hospitals, and the initial telephone contact point 116117 in 11 of 16 Federal States of Germany.

This cross-sectional study was based on a paper-based survey with healthcare professionals in both settings alongside the implementation of SmED.

### The intervention and its implementation

SmED is a computer-based decision support system which can be used by healthcare professionals (e.g. paramedics, nurses, practice assistants) for initial assessment as basis for demand management in outpatient emergency care services. SmED uses an algorithm based on the red flag approach to rate the urgency of need for medical treatment. The purpose of this software is to support health care professionals and to steer patients toward the right point of care based on their actual health needs. SmED is a certified medical product. A detailed description can be found in an earlier publication [8].

To facilitate the implementation of SmED educational workshops were organized for all Associations of Statutory Health Insurance Physicians of each participating Federal State of Germany. Participants were SmED-user in leading roles function as trainers for their colleagues. The trainers gave compressed educational workshops in their teams. A data protection concept, an implementation plan for each project site, and training concepts for potential users and trainers were developed. Moreover, quality management as well as a support management responsible for implementation sustainability were introduced by the project coordinator [8]. Nevertheless, the implementation into existing technical systems and workflows was highly variable at the time of this study and for organisational reasons not all study sites could start at the same time.

### Recruitment and study sample

All Associations of Statutory Health Insurance Physicians of each Federal State of Germany were invited to take part in this research project by the aQua-Institute for Applied Quality Improvement and Research in Health Care. 11 of 16 Federal States agreed to take part in the overall project, reasons for the non-participation of the remaining states were not provided. Potential participants were screened for eligibility by the Associations of Statutory Health Insurance Physicians. Healthcare professionals were eligible to participate if they were over the age of 18, have used the software, and gave consent to participate. The number of eligible healthcare professionals was reported to the research team at the University Hospital Heidelberg. According to this number information packages, including an invitation letter, an information leaflet, the paper-based questionnaire, and a reply envelope were put together and sent to a contact person responsible for distribution. The information sheet included contact details of research team members available to participants to discuss the study or address additional concerns or questions. Participants who decided to take part in the survey were requested to complete the questionnaire and post it directly to the researchers. Participation in the survey was voluntary.

### Questionnaire

The questionnaire was developed at the Department of General Practice and Health Services Research at the University Hospital Heidelberg, largely based on the *Consolidated Framework for Implementation Research* (CFIR). Based on the project aims and the CFIR, questions were formulated from the research team. The first section focused on sociodemographic characteristic such as age, gender, employment conditions, professional qualification, work setting, professional experience, and the use of SmED (*in categories*).

The second section compromised five scales which measured factors that may have an impact on the implementation process of SmED. When referring to all 5 scales, we will use the term Implementation of SmED in the following. The questionnaire design was guided by the CFIR but not further validated.

The first scale included 12 items related to the effectiveness/efficacy of the intervention being implemented (*Intervention Effectiveness/efficacy, 12 items*). This scale measures the practicability as well as completeness of SmED. This part included questions/statements such as: *Technical support was available at the beginning and during the implementation, the software has more advantages than disadvantages, or the software was adapted to my setting during the implementation and the effectiveness increased after the adaptions*. The second scale comprised items that covered the interprofessional context factors in relation to the implementation of SmED (*Interprofessional Context/ Occupational Interest, 8 items*) including questions like: *The collaboration between different healthcare professionals improved due to the implementation of SmED, I feel supported due to the interprofessional collaboration, or I think the implementation of SmED is successful/sustainable*. This scale measures the cooperation between physicians and users of SmED as well as the training concerning SmED. Scale three included items that covered factors of the individual context such as responsibilities or opinions regarding the implementation process and the software (*Individual Context, 13 items*). This scale measures the involvement of users in the implementation process of SmED as well as changes in their work routine. Statements or questions were for example *the value of my work increased due to the implementation of SmED, I am satisfied with the changes the implementation of SmED induced*. Scale four included items that measured organisational framework conditions (*Organisational Framework Conditions, 7 items*) including questions such as: *My workplace was ready for the implementation of SmED, the management supported and enhanced the implementation of SmED, or my workplace has enough resources to implement SmED*. This scale measures the support of the management level and the necessary resources for the implementation of SmED. The last scale focused on the medical context and included items covering questions *regarding the difference between the initial assessment by the software or the professionals, time needed per patient decrease due to the implementation of SmED, patients with a high urgency were identified faster and steered into the right point of care* (*Medical context, 9 questions*). This scale aims to measure the support of SmED concerning patient counselling and daily work routine.

All items of the 5 scales had five possible responses with scores ranging from 0 to 5; (5)*yes*, (4) *partly yes*, (3) *partly*, (2) *partly no*, (1) no *or not applicable/ I don´t know*. Specific threshold values were based on the equivalent partly yes or partly no on the Likert-Scale used for response. Therefore, mean scores could range from 1 to 5. Thus, mean scores above 3.0 indicated more positive norms regarding the Intervention Effectiveness/Efficacy, the Interprofessional Context/Occupational Interest, the Individual Context, the Organisational Framework Conditions, and the Medical Context in relation to the implementation process. Threshold of 3.0 for the mean scores for positive norms was chosen as it is more slightly over the middle of the range. The higher the individual mean scores the better was the implementation of SmED evaluated.

The questionnaire and all items per scale is attached as supplementary material (Additional file 1 and Additional file 2: Table S2 to S6 respectively).

### Data collection

According to the project coordinators in the participating regions, around 600 healthcare professionals used SmED at the beginning of the survey. Each eligible healthcare professional who used SmED at the time of the survey was invited to take part. Data collection was conducted between February 2020 and October 2020. Different strategies including e-mail reminder, telephone calls and the annual project coordination team meeting were used to maximize response rate.

### Data analysis

Data was analysed using the statistic software IBM SPSS Version 25.0. Mean scores for each scale (*Intervention Effectiveness/Efficacy, Implementation Process, Interprofessional Context/Occupational Interest, Individual Context, Organisational Framework Condition, and Medical Context*) were calculated, leaving out cases with missing values on more than a third of the items in a scale. This is how it is handled in questionnaires with a similar number of items per scale. Cronbach’s alpha was calculated to assess the internal consistency for each scale. A scale was considered as having a sufficiently internally consistency if Cronbach’s alpha was ≥ 0.70 (Additional file 2: Table S1, supplementary material).

Analysis of Variance (ANCOVA) was applied to examine significant differences in the mean scores of the five scales between the two settings (*Initial Telephone Contact Points 116,117 and Joint Counter*). Bivariate linear regression analysis was performed to assess associations of the five scales and demographic characteristic. Logistic regression analysis adjusted for gender and age was used to identify the relationship between the five scales and the use of SmED. *P* < 0.05 was considered significant in all analysis.

To assess agreement between participants we used the Intraclass correlation (ICC), which is used to assess agreement when there are two or more independent raters and the outcome is measured at a continuous level.

### Ethical consideration

Ethics approval was received by the medical ethics committee of the Medical Faculty of Heidelberg University in October 2018 (S-640/2018). Informed consent was obtained when healthcare professionals posted the completed questionnaire to the research team. Research conducted in this study was performed in accordance with the Declaration of Helsinki.

## Results

### Description of the study sample

In total 600 professionals were invited to take part, 200 completed the questionnaire and posted it to the research team (response-rate 33.3%). Table 1 shows that 63.1% of the healthcare professionals from the joint counter and 95.1% of the initial telephone contact point were female and older than 50 years old. 34.4% of the study participants from the initial telephone contact point and 76.8% from the joint counter were practice assistants. 42.0% of the professionals from the joint counter and 45.8% of the professionals from the initial telephone contact point were employed fulltime The majority of all participants had more than five years of professional experience, 50.4% from the initial telephone contact point and 66.7%from the joint counter. 32.1% of the professionals from the initial telephone contact point and 33.3% of the professionals from the Joint counter stated that they used SmED at every second patient encounter (Table 1).

**Table 1:** Characteristics of participants per out-of-hours service setting

N = 200 (100%)**	Initial telephone contact point n = 130 (65.5%)	Joint counter (Emergency department, outpatient emergency care service) n = 69 (34.5%)
*Age group*		
Between 18 and 29	22 (16.8)	8 (11.6)
Between 30 and 49	30 (22.9)	14 (20.3)
50 and older	76 (58.0)	46 (66.7)
No answer	3 (2.3)	1 (1.4)
*Sex*		
Female	82 (63.1)	66 (95.7)
Male	45 (34.6)	2 (2.9)
No answer	3 (2.3)	1 (1.4)
*Professional qualification*		
Physician	0	1 (1.4)
Nurse	10 (7.6)	3 (3.4)
Practice assistant	45 (34.4)	53 (76.8)
Emergency paramedic	31 (23.7)	2 (2.9)
Other	28 (21.4)	6 (8.7)
No answer	17 (13.0)	4 (5.8)
*Employment contract*		
Full-time (75–100%)	60 (45.8)	29 (42.0)
Part-time (50%)	20 (15.4)	10 (14.5)
Part-time (25%)	0	2 (2.9)
Temporary employment	37 (28.2)	24 (34.8)
Other	9 (6.9)	0
No answer	5 (3.8)	4 (5.8)
*Professional experience*		
between less than one year and two years	38 (29.9)	13 (18.8)
between two and four years	19 (14.5)	6 (8.7)
between four and five years	6 (4.6)	2 (2.9)
more than four years	66 (50.4)	46 (66.7)
No answer	2 (1.5)	2 (2.9)
*Use of SmED*		
every second patient	42 (32.1)	23 (33.3)
every third patient	36 (27.5)	8 (11.6)
every fourth patient	11 (8.4)	2 (2.9)
every fifth patient	5 (3.8)	2 (2.9)
I don't know	35 (26.7)	29 (42.0)
No answer	2 (1.5)	5 (7.2)

*Reported are frequencies and percentages n (%)

**One participant did not answer the question to affiliation to the out-of-hours service setting, the questionnaire was excluded from the description as well as the comparisons

Within ICC two-way random model—average measure was used. ICC (2,1) = 0.9 (95-CI = 0.88–0.92), based on the ICC we concluded that the test–retest reliability of this questionnaire is good.

### Evaluation of the implementation of SmED in the two settings

Participants in both settings evaluated the effectiveness/efficacy of SmED as moderate (mean score 2.97 SD 0.97 and mean score 2.95 SD 0.98), indicating that there is room for improvement in different areas of the effectiveness/efficacy of the software itself. No difference between the settings was found (*p* = 0.253). Participants from the initial telephone contact point indicated more positive norms regarding interprofessional context factors (3.01 (0.93)) compared to professionals working at the Joint Counter (2.64 (1.06)). However, the difference was not significant (*p* = 0.163). Individual Context factors such as: the implementation of SmED has enhanced the status of my work, the implementation of SmED has changed my workplace positively, or I am satisfied with the changes the implementation of SmED induced were evaluated as moderate by both groups (3.01 (0.88) and 3.05 (0.93)). No difference between the two settings was found (*p* = 0.503). The Organisational Framework Conditions (*e.g. organisational framework conditions have improved due to the implementation of SmED or my workplace has enough resources to implement SmED*) were evaluated moderately high by both settings (3.26 (1.16) and 3.53 (0.96)), indicating that the software has a positive impact on the workplace (*p* = 0.697). Both groups indicated relatively low mean scores when evaluating the Medical Context (e.g. *The initial assessments of SmED are coincided with my own assessments, with those of an earlier software, or with those by a physician*) in relation to SmED (2.54 (0.88) and 2.66 (0.87)). This indicates that the software may need to be improved based on the experiences of the healthcare professionals. No difference was found between the two settings (*p* = 0.637) (Table 2).

**Table 2:** User perceptions of the implementation of SmED at the initial telephone contact point and the Joint Counter

	Initial telephone contact point Mean (SD)	Joint counter mean (SD)	F	*P*-value
Intervention effectiveness/efficacy*	2.97 (0.97)	2.95 (0.98)	1.39	0.253
Interprofessional context/ occupational interest*	3.01 (0.93)	2.64 (1.06)	1.84	0.163
Individual context*	3.01 (0.88)	3.05 (0.93)	0.69	0.503
Organisational framework conditions*	3.26 (1.16)	3.53 (0.96)	0.36	0.697
Medical context*	2.54 (0.88)	2.66 (0.87)	0.45	0.637

*adjusted for gender and age, Minimum Score = 1, Maximum Score = 5, Scores < 3.0 indicates lower norms regarding the implementation of SmED

### Impact of the implementation of SmED on the use of SmED

Table 3 presents the results of the logistic regression analysis. Factors related to the Individual Context and to the Medical Context were associated with the reported use of SmED (*p* = 0.004 and 0.041, respectively). This indicates that a stronger perception of the presence of barriers for implementation related to the Individual Context are associated with a decrease by 0.535 units in the frequency of the use of SmED. Poorer evaluation of Medical Context factors was related to a decrease of 0.389 units in the frequency of the use of SmED. No other factors of the other scales had an impact on the use of SmED (Table 3).

**Table 3:** Use of SmED in relation to the implementation of SmED (Results of the logistic regression analysis)

Dependent predictor	B (SE)	Wald	Ex(B)	*p*-value
*Use of SmED*				
Intervention effectiveness/efficacy	0.315 (0.175)	3.24	0.73	0.072
Interprofessional context/occupational interest	− 0.288 (0.162)	7.91	0.75	0.076
Individual context	− 0.535 (0.185)	8.403	0.59	0.004*
Organisational framework conditions	− 0.297 (0.166)	6.60	0.74	0.074
Medical context	− 0.389 (0.190)	4.187	0.68	0.041*

### Association between individual characteristic and the implementation of SmED

Gender and age were associated with the Intervention Effectiveness/Efficacy scale. Healthcare professionals under the age of 29 evaluated factors concerning the effectiveness/efficacy of SmED slightly better compared to older professionals (b = 0.63, *p* = 0.010). Participants who identified as male tended to evaluate the effectiveness/efficacy of SmED slightly worse (b =  − 0.21, *p* = 0.042) (Table 4). This indicates that healthcare professionals who identified as male or older professionals perceived more disadvantages than advantages, that the software is not practicable (e.g. time requirement), or that SmED is too complex. Other characteristics were not significantly related to this scale (Table 4).

**Table 4:** Bivariate linear regression: correlates of individual characteristics with the intervention effectiveness/efficacy scale and the interprofessional context scale

Dependent predictor	B (SE)	Beta (β)	*p*-value
*Intervention effectiveness/efficacy scale*			
*Age*			
50 and older	*ref		
49 and younger	− 0.09 (0.15)	− 0.05	0.543
29 and younger	0.63 (0.24)	0.21	0.010*
*Gender*			
Female	*ref		
Male	− 0.21 (0.10)	− 0.15	0.042*
*Professional qualification*			
Practice assistant	*ref		
Nurse	0.60 (0.31)	0.14	0.061
Paramedic	− 0.216 (0.19)	− 0.08	0.277
Other	− 0.16 (0.20)	− 0.06	0.433
*Employment contract*			
(Full-time vs. Part-time/temporarily employment)	0.26 (0.15)	0.13	0.088
*Professional experience*			
(less than 5 years vs. more than 5 years)	− 0.18 (0.15)	− 0.09	0.230
*Use of SmED*			
(every second patient vs. every third to fifth patient)	− 0.28 (0.15)	− 0.14	0.070
*Out-of-hours service setting*			
Joint counter	*ref		
116,117	0.01 (0.16)	0.00	0.909
*Interprofessional context/ occupational interest scale*			
*Age*			
50 and older	*ref		
49 and younger	− 0.24 (0.15)	− 0.12	0.115
29 and younger	0.46 (0.21)	0.16	0.035*
*Gender*			
Female	*ref		
Male	− 0.17 (0.10)	− 0.12	0.091
*Professional qualification*			
Practice assistant	*ref		
Nurse	− 0.043 (0.30)	− 0.011	0.888
Paramedic	0.00 (0.20)	0.00	0.982
Other	0.03 (0.20)	0.01	0.865
*Employment contract*			
(Full-time vs. Part-time/ temporarily employment)	0.16 (0.15)	0.08	0.295
*Professional experience*			
(less than 5 years vs. more than 5 years)	− 0.32 (0.14)	− 0.15	0.032*
*Use of SmED*			
(every second patient vs. every third to fifth patient)	− 0.27 (0.15)	− 0.13	0.074
*Out-of-hours service setting*			
Joint counter	*ref		
116117	0.36 (0.15)	0.17	0.018*

*Significance level:* p* < 0.05

Age, professional experience, and out-of-hour-setting were related to the Interprofessional Context/Occupational Interest. Younger professionals evaluated Interprofessional Context/Occupational Interest scale slightly higher (b = 0.46, *p* = 0.035). Professionals working at the initial telephone contact points tended to evaluated the Interprofessional Context and its impact on the implementation of SmED slightly better (b = 0.36, *p* = 0.018). Participants with more professional experience tended to evaluated the Interprofessional Context and the implementation of SmED slightly worse compared to professionals with less years of professional experience (b =  − 0.32, *p* = 0.032). This indicates that professionals with few years of professional experience and participants of the initial telephone contact points perceived an improvement regarding the collaboration between different healthcare professionals. They stated that they think the implementation of SmED will be successful and sustainable. No other characteristics were associated with Interprofessional Context/Occupational Interest scale (Table 4).

Gender, age, professional experience, and frequency of use of SmED were associated with the Individual Context Scale. Participants who identified as male tended to evaluate the Individual Context slightly worse (b =  − 0.26, *p* = 0.005). Younger healthcare professionals tended to rate the Individual Context slightly better than their older colleagues (b = 0.41, *p* = 0.046). In addition, more experienced professionals rated the Individual Context Scale slightly worse (b =  − 0.28, *p* = 0.036). This shows that younger professionals felt that the value of their work had increased after the implementation of SmED, that their place of work changed in a positive way, and that they are satisfied with the changes the implementation of SmED induced. In contrast, healthcare professionals with more years of work experience reported to be overwhelmed by the changes the implementation of SmED induced. As shown in the logistic regression analysis, use of SmED was associated with the Individual Context (b =  − 0.41, *p* = 0.003). This indicates that if healthcare professionals evaluated the Individual Context worse frequency of use of SmED decreased. Other factors were not significant (Table 5).

**Table 5:** Bivariate linear regression: correlates of individual characteristics with the Individual Context Scale and the Organisational Framework Conditions Scale

Dependent predictor	B (SE)	Beta (β)	*p*-value
*Individual context scale*			
*Age*			
50 and older	*ref		
49 and younger	− 0.13 (0.14)	− 0.07	0.384
29 and younger	0.41 (0.20)	0.16	0.046*
*Gender*			
Female	*ref		
Male	− 0.26 (0.09)	− 0.21	0.005*
*Professional qualification*			
Practice assistant	*ref		
Nurse	0.22 (0.26)	0.06	0.400
Paramedic	− 0.10 (0.18)	− 0.04	0.581
Other	0.09 (0.18)	0.04	0.622
*Employment contract*			
(Full-time vs. Part-time/temporarily employment)	0.03 (0.14)	0.02	0.818
*Professional experience*			
(less than 5 years vs. more than 5 years)	− 0.28 (0.13)	− 0.15	0.036*
*Use of SmED*			
(every second patient vs. ever third to fifth patient)	− 0.41 (0.14)	− 0.22	0.003*
*Out-of-hours service setting*			
Joint counter	*ref		
116,117	− 0.05 (0.14)	− 0.03	0.727
*Organisational framework conditions scale*			
*Age*			
50 and older	*ref		
49 and younger	− 0.19 (0.16)	− 0.09	0.243
29 and younger	0.37 (0.24)	0.12	0.126
*Gender*			
Female	*ref		
Male	− 0.15 (0.11)	− 0.10	0.176
*Professional qualification*			
Practice assistant	*ref		
Nurse	0.01 (0.31)	0.00	0.970
Paramedic	− 0.08 (0.20)	− 0.03	0.685
Other	0.07 (0.22)	0.02	0.735
*Employment contract*			
(Full-time vs. part-time/temporarily employment)	− 0.06 (0.16)	− 0.03	0.703
*Professional experience*			
(less than 5 years vs. more than 5 years)	− 0.53 (0.15)	− 0.25	0.001*
*Use of SmED*			
(every second patient vs. ever third to fifth patient)	− 0.29 (0.16)	− 0.13	0.073
*Out-of-hours service setting*			
Joint counter	*ref		
116117	0.27 (0.16)	− 0.12	0.099

*Significance level:* p* < 0.05

The only factor associated with Organisational Framework Conditions was the professional experience (b =  − 0.53, *p* = 0.001). Participants with more professional experience tended to evaluate the Organisational Framework Conditions during the implementation of SmED slightly worse. This indicates that younger professionals with only a few years of professional experience appreciated the support of the management. Younger professionals more likely stated that the management supported and facilitated the implementation of SmED, and that enough resources were available. In addition, they perceived the implementation of SmED as useful and reasonable (Table 5).

Various individual characteristics showed a significant association with the Medical Context of the implementation of SmED. Participants under the age of 29 evaluated the Medical Context slightly better than their older colleagues (b = 0.44, *p* = 0.038). Participants with more than five years of professional experience and who identified as male tended to evaluate the Medical Context within the implementation of SmED slightly worse (b =  − 0.29, *p* = 0.038 and b =  − 0.21, *p* = 0.028, respectively). This indicates that, younger professionals with only few years of professional experience and female healthcare professionals perceived SmED as a useful tool in patient counselling and stated that SmED reduced time needed per patient contact. Furthermore, those participants who used SmED within every third to fifth patient rated the Medical Context slightly worse compared to their colleagues who used SmED within every second patient (b = -0.29,   *p* = 0.038). This indicates that participants with a frequent use of SmED evaluated the Medical Context of SmED better than participants using SmED infrequently (Table 6).

**Table 6:** Bivariate linear regression: correlates of individual characteristics with the Medical Context scale

Dependent predictor	B (SE)	Beta (β)	*p*-value
*Medical context scale*			
*Age*			
50 and older	*ref		
49 and younger	− 0.07 (0.14)	− 0.41	0.614
29 and younger	0.44 (0.21)	0.17	0.038*
*Gender*			
Female	*ref		
Male	− 0.21 (0.09)	− 0.17	0.028*
*Professional qualification*			
Practice assistant	*ref		
Nurse	0.15 (0.25)	0.05	0.552
Paramedic	− 0.15 (0.17)	− 0.07	0.288
Other	0.14 (0.19)	0.06	0.474
*Employment contract*			
(Full-time vs. Part-time/temporarily employment)	0.22 (0.14)	0.12	0.117
*Professional experience*			
(less then 5 years vs. more than 5 years)	− 0.28 (0.14)	− 0.16	0.042*
*Use of SmED*			
(every second patient vs. ever third to fifth patient)	− 0.29 (0.13)	− 0.16	0.038*
*Out-of-hours service setting*			
Joint counter	*ref		
116117	− 0.13 (0.14)	− 0.07	0.352

*Significance level:* p* < 0.05

## Discussion

Although, assessment of complex interventions in different settings and locations with different working cultures and technical environments is rather demanding, the aim of this study was to evaluate the implementation of SmED, a computer-based decision support system which can be used in out-of-hours service settings for initial assessment as basis for demand management, from the point of view of the users in order to facilitate uptake and sustainable implementation. Therefore, questionnaires from 200 users of SmED were evaluated in order to access the frequency of use of the software, general perceptions and experience of users during the implementation process, and to detect potential associations between the views on the uptake of SmED, on reported use of SmED, and individual characteristics.

### Principal findings

Several factors of the Individual Context and Medical Context, as perceived by the users, were associated with the frequency of use of SmED. Several individual characteristics showed association with the five scales of implementation of SmED. Female and younger participants and participants with less than five years of professional experience evaluated the implementation as more positive. The Individual Context and frequency of use of SmED were associated. This indicates that health professionals who were not satisfied with their individual context factors (e.g. *I have been able to share my opinion at the beginning of the implementation of the software or my work routine has negatively changed since the implementation of the software)* during the implementation of SmED, did not use the software within every second patient. To increase the use of SmED, healthcare professionals should be involved in implementation process in a more comprehensive way. Furthermore, older healthcare professionals and professionals with more professional experience should be more encouraged and involved in the implementation process.

### Comparison with prior work

Porter et al. [15] found out that organisational and technological readiness for innovation is an important factor that influences the success of the implementation process. In this study, organisational factors related to the implementation process were evaluated moderately positive by the software users which could have had a positive impact on the implementation process and therefore the sustainability of the implementation of SmED. However, user perception of the intervention effectiveness/efficacy showed room for improvement. The implementation of innovative technology within the healthcare sector can meet with resistance from healthcare professionals [13]. In this study, factors related to the individual context such as *I have been able to share my opinion prior to the beginning, at the beginning, and during the implementation of SmED* were evaluated to a moderate degree. This may have an impact on the success of the implementation process. Thus, it is important to consider individual opinions of user, in this case healthcare professionals, when implementing a new software to enhance implementation sustainability. In addition, a review regarding clinical decision support systems (CDSS) for ED triage conducted by Fernandes et al. [16] found out that in more than half of the studies the implementation phase seemed to lack.

Kyratsisv et al. [13] stated that it is important to train user in how to use technological innovations and adapt the innovations to a certain setting early during the implementation process in order to facilitate sustainable implementation. Effectiveness/Efficacy of the Intervention and factors related to the Interprofessional Context/Occupational Interest were rated as moderate in this study. This may indicate that technical support prior and during the implementation process may have been unsatisfactorily for some professionals and that interprofessional collaboration still needs to be improved. Although the difference was not significant healthcare professionals at the Joint Counter evaluated factors related to the Effectiveness/Efficacy of the Intervention or the Interprofessional Context/Occupational Interest slightly worse, this may indicate that the software was not sufficient adopted to their setting or that the training was not satisfactorily.

According to Porter et al. [15] support by management and local leaders is an important factor regarding the success of the implementation process. The results of this study showed that participants rated the Organisational Framework Conditions moderately high indicating that they felt supported by their management and valued this support which in turn may influence the implementation process positively.

Professional experience predicted Interprofessional Context/ Occupational Interest Scale, Individual Context Scale, Organisational Framework Conditions Scale and Medical Context Scale indicating that health care professionals with more experience were less satisfied with the implementation of SmED. These results are in line with those of Forsegren et al. [17]. They conducted a study in Sweden in 2009 on Job satisfaction in nursing and working with Manchester Triage. The experienced triage nurses in particular perceived Manchester Triage as “rigid” and felt that it needed development. Forsegren et al. [17] have also shown that competent nurse feel that they could act independently successfully. Which may also be the feeling of healthcare professionals working with SmED. Another explanation for the dissatisfaction with SmED in health care professionals with more work experience could be the fear of getting replaced by it or an assumption that their professional experience is not valued, we assume.

20.3% of the participating healthcare professionals reported that patients with a high degree of urgency have been identified faster due to the use of SmED, support regarding patient consulting was helpful for 23.9% of all healthcare professionals, and results of SmED regarding urgency and severity were only partly consistent with those by a physician (reported by 19.4% of all professionals) or the healthcare professionals themselves (reported by 30.5%). These results are in line with those by Egbunike et al. [18]. They conducted a study in the United Kingdom on the efficiency of a triage system used in GP out-of-hours settings. A major concern indicated by the triage users were long waiting times for patients and a general inefficiency of the system [18]. These results indicated that user feedback, particularly on the points mention above, should integrated into implementation process in order to improve the efficiency of the software.

## Limitations

The study has some limitations. Despite repeated reminders to increase response rate only 33.3% of eligible participants was reached. One reason could be the voluntary participation, as well as it cannot be assured that every potential participant received a questionnaire. Furthermore, data collection was interrupted during the SARS-CoV-2 pandemic between April 2020 and August 2020 because some of the settings decided to stop using SmED during the ongoing pandemic due to an increase of workload caused by non-medical requests of patients. Another limitation was that regulation regarding the use of SmED differed between the regions. In some setting it was mandatory to use SmED frequently, in other settings on the other hand health care professionals could decide when and if they use SmED. Furthermore, the quality of educational workshops may have differed between the regions, due to some trainers modifying the workshops. This may had an impact on the frequency of use of SmED and on the overall perceptions regarding the implementation process. The questionnaire was completed based on self-report: thus, results might be biased by common bias such as method variance, self-report, selection, or social desirability bias. Moreover, we do not know when healthcare professionals filled in the survey. They may were on duty, therefore responses could be influenced by events on the day or workload. Hence, the survey should be repeated to evaluate whether the results differ in terms of day and shift. Sampling bias regarding profession, age and healthcare experience cannot be excluded. SmED was mainly developed to be used at the initial contact point 116,117 and not at the Joint Counter. This may had an impact on the perceptions and experiences of the users particularly at the Joint Counter. An version which can be use at the Joint Counter is currently being developed. The cross-sectional design only allows to identify association but causal interference cannot be made. Thus, the results have to be interpreted with caution.

### Future research

Future research should be conducted after fully integration of SmED into the technical frameworks used at the different locations and after the development of the version which can be used at the Joint Counter. The aim of SmED is support health care professionals to access the severity and urgency of presented health issues and to reduce the number of unnecessary ED visits, thus research is needed to evaluate if the number of unnecessary ED visits decreased after the implementation of SmED.

## Conclusion

This study provides useful information for the implementation of a standardized medical initial assessment for outpatient emergency care services in Germany. We found that especially younger healthcare professionals tend to evaluate SmED and the implementation process slightly better, whereas healthcare professionals with more than five years of experience were less satisfied with SmED and its implementation process. It is important to include healthcare professionals, particularly more experienced professionals, in the implementation process in order to facilitate sustainable implementation. In addition, training of potential user prior and during the implementation process and the adaption of organisational context factors is crucial.

## Supplementary Information

**Additional file 1**: Questionnaire – Demand/ SmED user survey.

**Additional file 2**: Table S1: Results of the reliability analysis. Table S2 to S6: Items of the different scores and frequency of answers per setting.

## Data Availability

The dataset generated and analysed during the current study will not be made publicly available due to European Data Protection Law but maybe available by the corresponding author on reasonable request.

## Abbreviations

aQua: Institute for Applied Quality Improvement and Research in Health Care
EDs: Emergency Departements
SmED: Standardisierte medizinische Ersteinschätzung in Deutschland (Engl. Standardized medical initial assessment in Germany)
