# Supplementary material for: Enhancing implementation of a standardized initial assessment for demand management in outpatient emergency care in Germany: a quantitative process evaluation
Source: BMC Med Inform Decis Mak. 2021 Nov 16;21:318. doi: 10.1186/s12911-021-01685-6 (PMC8592824; doi:10.1186/s12911-021-01685-6)
Supplement: Supplementary file 1 — Additional file 1: Questionnaire – Demand/ SmED user survey. [file 12911_2021_1685_MOESM1_ESM.docx]

**Enhancing implementation of a standardized initial assessment for demand management in outpatient emergency care in Germany: a quantitative process evaluation**

Amanda Breckner^1*^, Catharina Roth^1*^, Joachim Szecsenyi^1^, Michel Wensing^1^

1 Heidelberg University Hospital, Department of General Practice and Health Services Research. Marsilius Arcades, West Tower, Im Neuenheimer Feld 130, 69120 Heidelberg, Germany.

Corresponding Author:

Amanda Breckner*, Department of General Practice and Health Services Research, Heidelberg University Hospital, Marsilius Arcades, West Tower, Im Neuenheimer Feld 130, 69120 Heidelberg, Germany

[Amanda.breckner@med.uni-heidelberg.de](mailto:Amanda.breckner@med.uni-heidelberg.de), 0049 6221 56 34646

Co-Authors:

Catharina Roth*, Department of General Practice and Health Services Research, Heidelberg University Hospital

Joachim Szecsenyi, Department of General Practice and Health Services Research, Heidelberg University Hospital

Michel Wensing, Department of General Practice and Health Services Research, Heidelberg University Hospital

*****These authors contributed equally

**Additional file 1: Questionnaire – Demand/ SmED user survey**

Note:

This questionnaire was developed for this study and has not been published elsewhere. It consists of non-validated questions. The original language of the questionnaire was German and was translated into English language through one-way translation.

**Professional experience and demographics**

| What age group do you belong to? |
| --- |
| 18 to 24 years / 25 to 29 years / 30 to 39 years / 40 to 49 years / 50 to 59 years / 60 and older / no answer |
| Which gender can you identify with? |
| Female / male / non-binary / other / no answer |
| Which acquisition situation suits you? |
| Full-time job (100 %) / part-time job (50 %) / part-time job (25 %) / part-time job (75%) / temporary employment / other / no answer |
| Professional Qualification |
| Physician / Nurse / Practice assistant / emergency paramedic / other / no answer |
| In which out-of-hours service setting are you working? |
| Initial telephone contact point / Joint counter |
| How much professional experience do you have in the context out-of-hours service? |
| Less then one year/ between one and two years/ between two and three years/ between three and fours years/ between four and five years/ more than five years |
| SmED was used approx. within … |
| Every second patient / every third patient / every fourth patient / every fifth patient / I don’t know / no answer |

**Intervention Effectiveness/Efficacy**

| The software has more advantages than disadvantages for the user |
| --- |
| Yes / Partly yes / Partly / Partly no / No / I don´t know, no answer |
| The new software has more advantages than disadvantages compared to the software used before |
| Yes / Partly yes / Partly / Partly no / No / I don´t know, no answer |
| There was a technical contact person **prior to the implementation** of the software |
| Yes / Partly yes / Partly / Partly no / No / I don´t know, no answer |
| There was a technical contact person **at the beginning of the implementation** of the software |
| Yes / Partly yes / Partly / Partly no / No / I don´t know, no answer |
| There was a technical contact person **during the implementation** of the software |
| Yes / Partly yes / Partly / Partly no / No / I don´t know, no answer |
| The software was adapted to my setting **during the implementation** |
| Yes / Partly yes / Partly / Partly no / No / I don´t know, no answer |
| The efficiency of the software increased after the adaption |
| Yes / Partly yes / Partly / Partly no / No / I don´t know, no answer |
| The software is practicable and easy to use |
| Yes / Partly yes / Partly / Partly no / No / I don´t know, no answer |
| The software is easy to use and understand |
| Yes / Partly yes / Partly / Partly no / No / I don´t know, no answer |
| The software has been used during all patient contacts |
| Yes / Partly yes / Partly / Partly no / No / I don´t know, no answer |
| All medical and consulting services are included in the software |
| Yes / Partly yes / Partly / Partly no / No / I don´t know, no answer |
| The improvements the implementation of the software was meant to bring are the same improvements I can see during my work routine |
| Yes / Partly yes / Partly / Partly no / No / I don´t know, no answer |

**Interprofessional Context/ Occupational Interest**

| The collaboration between different profession has (sustainably) improve due to the implementation of the software |
| --- |
| Yes / Partly yes / Partly / Partly no / No / I don´t know, no answer |
| Employees of the emergency department have been disburdened due to the improved steering of patients |
| Yes / Partly yes / Partly / Partly no / No / I don´t know, no answer |
| I feel supported by the interprofessional collaboration |
| Yes / Partly yes / Partly / Partly no / No / I don´t know, no answer |
| I think the implementation of the software is successful |
| Yes / Partly yes / Partly / Partly no / No / I don´t know, no answer |
| I think the implementation of the software is sustainable |
| Yes / Partly yes / Partly / Partly no / No / I don´t know, no answer |
| There was a training prior to the implementation of the software |
| Yes / Partly yes / Partly / Partly no / No / I don´t know, no answer |
| The training was useful and it was helpful during the use of the software |
| Yes / Partly yes / Partly / Partly no / No / I don´t know, no answer |
| During the implementation of the software other trainings which have been helpful were conducted |
| Yes / Partly yes / Partly / Partly no / No / I don´t know, no answer |

**Individual Context**

| The implementation of the software has made work more valuable |
| --- |
| Yes / Partly yes / Partly / Partly no / No / I don´t know, no answer |
| I am actively involved in the success of the implementation of the software |
| Yes / Partly yes / Partly / Partly no / No / I don´t know, no answer |
| I don’t have a problem with adapting my work routine to the implementation of the software and the use of the software |
| Yes / Partly yes / Partly / Partly no / No / I don´t know, no answer |
| I have been included actively in the implementation of the software from beginning of the project until now |
| Yes / Partly yes / Partly / Partly no / No / I don´t know, no answer |
| My area of responsibility has **increased** since the implementation of the software |
| Yes / Partly yes / Partly / Partly no / No / I don´t know, no answer |
| My area of responsibility has **decreased** since the implementation of the software |
| Yes / Partly yes / Partly / Partly no / No / I don´t know, no answer |
| My work routine has **positively changed** since the implementation of the software |
| Yes / Partly yes / Partly / Partly no / No / I don´t know, no answer |
| My work routine has **negatively changed** since the implementation of the software |
| Yes / Partly yes / Partly / Partly no / No / I don´t know, no answer |
| I am **satisfied with the changes** the implementation of the software induced |
| Yes / Partly yes / Partly / Partly no / No / I don´t know, no answer |
| I am **overstrained due to the changes** the implementation of the software induced |
| Yes / Partly yes / Partly / Partly no / No / I don´t know, no answer |
| I have been able to share my opinion **prior to** **the beginning of the implementation** of the software |
| Yes / Partly yes / Partly / Partly no / No / I don´t know, no answer |
| I have been able to share my opinion at **the beginning of the implementation** of the software |
| Yes / Partly yes / Partly / Partly no / No / I don´t know, no answer |
| I have been able to share my opinion **during the implementation** of the software |
| Yes / Partly yes / Partly / Partly no / No / I don´t know, no answer |

**Organisational Framework Conditions**

| My workplace was ready for the implementation of the software |
| --- |
| Yes / Partly yes / Partly / Partly no / No / I don´t know, no answer |
| My workplace had enough resources for the implementation of the software |
| Yes / Partly yes / Partly / Partly no / No / I don´t know, no answer |
| The organisational framework conditions supported the implementation of the software |
| Yes / Partly yes / Partly / Partly no / No / I don´t know, no answer |
| The management level supported the implementation of the software |
| Yes / Partly yes / Partly / Partly no / No / I don´t know, no answer |
| I value the support of the management level |
| Yes / Partly yes / Partly / Partly no / No / I don´t know, no answer |
| I think the implementation of the software is reasonable and justified |
| Yes / Partly yes / Partly / Partly no / No / I don´t know, no answer |
| Organisational framework conditions changed positive due to the implementation of the software (e.g. saving of time) |
| Yes / Partly yes / Partly / Partly no / No / I don´t know, no answer |

**Medical Context**

| The initial assessments of the software are coincided with my own assessments or with those of an earlier software |
| --- |
| Yes / Partly yes / Partly / Partly no / No / I don´t know, no answer |
| The initial assessments of the software are coincided with the assessment of an physician |
| Yes / Partly yes / Partly / Partly no / No / I don´t know, no answer |
| Patients with a high degree of urgency have been identified faster due to the software |
| Yes / Partly yes / Partly / Partly no / No / I don´t know, no answer |
| Patients with a low degree of urgency have been identified due to the software and were steered into the right point of care |
| Yes / Partly yes / Partly / Partly no / No / I don´t know, no answer |
| The support by the software regarding patient counselling was useful |
| Yes / Partly yes / Partly / Partly no / No / I don´t know, no answer |
| I think the implementation of the software may increases risk of patient harm |
| Yes / Partly yes / Partly / Partly no / No / I don´t know, no answer |
| Patient satisfaction increased due to easier access to medical resources |
| Yes / Partly yes / Partly / Partly no / No / I don´t know, no answer |
| The use of the software reduced time needed per patient |
| Yes / Partly yes / Partly / Partly no / No / I don´t know, no answer |
| There was a contact person available in case of uncertainties regarding the medical assessment |
| Yes / Partly yes / Partly / Partly no / No / I don´t know, no answer |
