# Supplementary material for: Enhancing implementation of a standardized initial assessment for demand management in outpatient emergency care in Germany: a quantitative process evaluation
Source: BMC Med Inform Decis Mak. 2021 Nov 16;21:318. doi: 10.1186/s12911-021-01685-6 (PMC8592824; doi:10.1186/s12911-021-01685-6)
Supplement: Supplementary file 2 — Additional file 2: Table S1: Results of the reliability analysis. Table S2 to S6: Items of the different scores and frequency of answers per setting. [file 12911_2021_1685_MOESM2_ESM.docx]

**Enhancing implementation of a standardized initial assessment for demand management in outpatient emergency care in Germany: a quantitative process evaluation**

Amanda Breckner^1*^, Catharina Roth^1*^, Joachim Szecsenyi^1^, Michel Wensing^1^

1 Heidelberg University Hospital, Department of General Practice and Health Services Research. Marsilius Arcades, West Tower, Im Neuenheimer Feld 130, 69120 Heidelberg, Germany.

Corresponding Author:

Amanda Breckner*, Department of General Practice and Health Services Research, Heidelberg University Hospital, Marsilius Arcades, West Tower, Im Neuenheimer Feld 130, 69120 Heidelberg, Germany

[Amanda.breckner@med.uni-heidelberg.de](mailto:Amanda.breckner@med.uni-heidelberg.de), 0049 6221 56 34646

Co-Authors:

Catharina Roth*, Department of General Practice and Health Services Research, Heidelberg University Hospital

Joachim Szecsenyi, Department of General Practice and Health Services Research, Heidelberg University Hospital

Michel Wensing, Department of General Practice and Health Services Research, Heidelberg University Hospital

*****These authors contributed equally

# Additional file 2: Tables S1 to S6

Table S1: Results of the reliability analysis

Table S2 to S6: Items of the different scores and frequency of answers per setting

Table S1: Results of the reliability analysis

|  | **N** | **Number of items** | **Answering categories** | **Scale construction** | **Cronbach’s alpha** |
| --- | --- | --- | --- | --- | --- |
| Implementation process | 199 | 12 | 5 | Average | 0.79 |
| Interprofessional context | 199 | 8 | 5 | Average | 0.68 |
| Individual context | 200 | 13 | 5 | Average | 0.84 |
| Organisational framework conditions | 198 | 7 | 5 | Average | 0.75 |
| Medical Context | 198 | 9 | 5 | Average | 0.74 |

Table S2: Intervention Effectiveness/Efficacy

|  | **Initial telephone contact point (116117)**  **n=130 (65.5%)** | **Joint Counter**  **n=69 (34.5%)** |
| --- | --- | --- |
| The software has more advantages than disadvantages for the user | | |
| Yes | 42 (32.1) | 12 (17.4) |
| Partly | 34 (26.0) | 19 (27.5) |
| No | 54 (41.2) | 36 (52.2) |
| I don’t know/ no answer | 1 (0.8) | 2 (2.9) |
| The new software has more advantages than disadvantages compared to the software used before | | |
| Yes | 31 (23.7) | 10 (14.5) |
| Partly | 21 (16.0) | 10 (14.5) |
| No | 54 (41.2) | 32 (46.4) |
| I don’t know/ no answer | 25 (19.1) | 17 (24.6) |
| There was a technical contact person **prior to the implementation** of the software | | |
| Yes | 56 (42.7) | 29 (42.6) |
| Partly | 8 (6.1) | 8 (11.8) |
| No | 27 (20.6) | 10 (14.7) |
| I don’t know/ no answer | 40 (30.5) | 21 (30.9) |
| There was a technical contact person **at the beginning of the implementation** of the software | | |
| Yes | 72 (55.0) | 31 (45.6) |
| Partly | 11 (8.4) | 6 (8.8) |
| No | 15 (11.5) | 9 (13.2) |
| I don’t know/ no answer | 33 (25.2) | 22 (32.4) |
| There was a technical contact person **during the implementation** of the software | | |
| Yes | 76 (58.0) | 32 (47.1) |
| Partly | 15 (11.5) | 6 (88.8) |
| No | 16 (12.2) | 11 (16.2) |
| I don’t know/ no answer | 24 (18.3) | 19 (27.9) |
| The software was adapted to my setting **during the implementation** | | |
| Yes | 42 (32.1) | 15 (21.7) |
| Partly | 23 (17.6) | 12 (17.4) |
| No | 38 (29.0) | 12 (17.4) |
| I don’t know/ no answer | 28 (21.4) | 30 (43.5) |
| The efficiency of the software increased after the adaption | | |
| Yes | 44 (33.6) | 18(26.1) |
| Partly | 22 (16.8) | 9 (13.0) |
| No | 39 (29.8) | 16 (32.2) |
| I don’t know/ no answer | 26 (19.8) | 26 (37.7) |
| The software is practicable and easy to use | | |
| Yes | 28 (21.4) | 12 (17.4) |
| Partly | 24 (18.3) | 9 (13.0) |
| No | 73 (55.7) | 47 (68.1) |
| I don’t know/ no answer | 6 (4.6) | 1 (1.4) |
| The software is easy to use and understand | | |
| Yes | 81 (61.8) | 38 (55.1) |
| Partly | 24 (18.3) | 16 (32.2) |
| No | 22 (16.8) | 14 (20.3) |
| I don’t know/ no answer | 4 (3.1) | 1 (1.4) |
| The software has been used during all patient contacts | | |
| Yes | 43 (32.8) | 26 (37.7) |
| Partly | 33 (25.2) | 13 (18.8) |
| No | 43 (32.8) | 23 (33.3) |
| I don’t know/ no answer | 12 (9.2) | 7 (10.1) |
| All medical and consulting services are included in the software | | |
| Yes | 19 (14.5) | 15 (21.7) |
| Partly | 21 (16.0) | 21 (30.4) |
| No | 87 (66.4) | 27 (39.1) |
| I don’t know/ no answer | 4 (3.1) | 6 (8.7) |
| The improvements the implementation of the software was meant to bring are the same improvements I can see during my work routine | | |
| Yes | 20 (15.3) | 9 (13.0) |
| Partly | 30 (22.9) | 10 (14.5) |
| No | 61 (46.6) | 39 (56.5) |
| I don’t know/ no answer | 20 (15.3) | 11 (15.9) |

Table S3: Interprofessional Context/ Occupational Interest

|  | **Initial telephone contact point (116117)**  **n=130 (65.5%)** | **Joint Counter**  **n=69 (34.5%)** |
| --- | --- | --- |
| The collaboration between different profession has (sustainably) improve due to the implementation of the software | | |
| Yes | 20 (15.3) | 9 (13.0) |
| Partly | 21 (16.0) | 5 (7.2) |
| No | 45 (34.4) | 35 (50.7) |
| I don’t know/ no answer | 45 (34.4) | 20 (29.0) |
| Employees of the emergency department have been disburdened due to the improved steering of patients | | |
| Yes | 7 | 10 (14.5) |
| Partly | 8 | 9 (13.0) |
| No | 27 | 31 (44.9) |
| I don’t know/ no answer | 88 | 19 (27.5) |
| I feel supported by the interprofessional collaboration | | |
| Yes | 27 (20.6) | 13 (18.8) |
| Partly | 32 (24.4) | 13 (18.8) |
| No | 50 (38.2) | 37 (53.6) |
| I don’t know/ no answer | 22 (16.8) | 6 (8.7) |
| I think the implementation of the software is successful | | |
| Yes | 30 (22.9) | 15 (21.7) |
| Partly | 29 (22.1) | 10 (14.5) |
| No | 60 (45.8) | 43 (62.3) |
| I don’t know/ no answer | 12 (9.2) | 1 (1.4) |
| I think the implementation of the software is sustainable | | |
| Yes | 29 (22.1) | 13 (18.8) |
| Partly | 30 (22.9) | 11 (15.9) |
| No | 56 (42.7) | 34 (49.3) |
| I don’t know/ no answer | 16 (12.2) | 11 (15.9) |
| There was a training prior to the implementation of the software | | |
| Yes | 100 (76.3) | 45 (65.2) |
| Partly | 13 (9.9) | 8 (11.6) |
| No | 14 (10.7) | 12 (17.4) |
| I don’t know/ no answer | 4 (3.1) | 4 (5.8) |
| The training was useful and it was helpful during the use of the software | | |
| Yes | 95 (72.5) | 43 (62.3) |
| Partly | 21 (16.0) | 13 (18.8) |
| No | 12 (9,2) | 8 (11.6) |
| I don’t know/ no answer | 3 (2.3) | 5 (7.2) |
| During the implementation of the software other trainings which have been helpful were conducted | | |
| Yes | 33 (25.2) | 11 (15.9) |
| Partly | 17 (13.0) | 5 (7.2) |
| No | 73 (55.7) | 51 (73.9) |
| I don’t know/ no answer | 8 (6.1) | 2 (2.9) |

Table S4: Individual Context

|  | **Initial telephone contact point (116117)**  **n=130 (65.5%)** | **Joint Counter**  **n=69 (34.5%)** |
| --- | --- | --- |
| The implementation of the software has made work more valuable | | |
| Yes | 22 (16.8( | 8 (11.6) |
| Partly | 16 /12.2) | 7 (10.1) |
| No | 88 (67.2) | 50 (72.5) |
| I don’t know/ no answer | 5 (3.8) | 4 (5.8) |
| I am actively involved in the success of the implementation of the software | | |
| Yes | 67 (51.1) | 43 (62.3) |
| Partly | 28 (21.4) | 14 (20.3) |
| No | 24 (18.3) | 6 (8.7) |
| I don’t know/ no answer | 12 (9.2) | 6 (8.7) |
| I don’t have a problem with adapting my work routine to the implementation of the software and the use of the software | | |
| Yes | 73 (55.7) | 27 (39.1) |
| Partly | 22 (16.8) | 21 (30.4) |
| No | 33 (25.2) | 20 (29.) |
| I don’t know/ no answer | 3 (2.3) | 1 (1.4) |
| I have been included actively in the implementation of the software from beginning of the project until now | | |
| Yes | 34 (26.0) | 29 (42.0) |
| Partly | 20 (15.3) | 8 (11.6) |
| No | 56 (42.7) | 22 (31.9) |
| I don’t know/ no answer | 21 (16.0) | 10 (14.5) |
| My area of responsibility has **increased** since the implementation of the software | | |
| Yes | 24 (18.3) | 12 (17.4) |
| Partly | 10 (7.6) | 9 (13.0) |
| No | 80 (61.1) | 42 (60.9) |
| I don’t know/ no answer | 17 (13.0) | 6 (8.7) |
| My area of responsibility has **decreased** since the implementation of the software | | |
| Yes | 20 (15.3) | 8 (11.6) |
| Partly | 11 (8.4) | 4 (5.8) |
| No | 81 (61.8) | 46 (66.7) |
| I don’t know/ no answer | 19 (14.5) | 11 (15.9) |
| My work routine has **positively changed** since the implementation of the software | | |
| Yes | 17 (13.0) | 8 (11.6) |
| Partly | 24 (18.3) | 11 (15.9) |
| No | 76 (58.0) | 47 (68.1) |
| I don’t know/ no answer | 14 (10.7) | 3 (4.3) |
| My work routine has **negatively changed** since the implementation of the software | | |
| Yes | 50 (38.2) | 21 (30.4) |
| Partly | 22 (16.8) | 8 (11.6) |
| No | 48 (36.6) | 35 (50.7) |
| I don’t know/ no answer | 11 (8.4) | 5 (7.2) |
| I am **satisfied with the changes** the implementation of the software induced | | |
| Yes | 32 (24.4) | 10 (14.5) |
| Partly | 29 (22.1) | 18 (26.1) |
| No | 63 (48.1) | 38 (55.1) |
| I don’t know/ no answer | 7 (5.3) | 3 (4.3) |
| I am **overstrained due to the changes** the implementation of the software induced | | |
| Yes | 4 (3.1) | 5 (7.2) |
| Partly | 11 (8.4) | 11 (15.9) |
| No | 108 (82.4) | 49 (71.0) |
| I don’t know/ no answer | 8 (6.1) | 4 (5.8) |
| I have been able to share my opinion **prior to** **the beginning of the implementation** of the software | | |
| Yes | 41 (31.3) | 20 (29.0) |
| Partly | 14 (10.7) | 4 (5.8) |
| No | 55 (42.0) | 34 (49.3) |
| I don’t know/ no answer | 21 (16.0) | 11 (15.9) |
| I have been able to share my opinion at **the beginning of the implementation** of the software | | |
| Yes | 51 (38.9) | 28 (40.6) |
| Partly | 19 (14.5) | 9 (13.0) |
| No | 45 (34.4) | 25 (36.2) |
| I don’t know/ no answer | 16 (12.2) | 7 (10.1) |
| I have been able to share my opinion **during the implementation** of the software | | |
| Yes | 67 (51.1) | 39 (56.5) |
| Partly | 18 (13.7) | 7 (10.1) |
| No | 35 (26.7) | 19 (27.5) |
| I don’t know/ no answer | 11 (8.4) | 4 (5.8) |

Table S5: Organisational Framework Conditions

|  | **Initial telephone contact point (116117)**  **n=130 (65.5%)** | **Joint Counter**  **n=69 (34.5%)** |
| --- | --- | --- |
| My workplace was ready for the implementation of the software | | |
| Yes | 95 (72.5) | 44 (63.8) |
| Partly | 16 (12.2) | 4 (5.8) |
| No | 11 (8.4) | 12 (17.4) |
| I don’t know/ no answer | 9 (6.9) | 9 (13.0) |
| My workplace had enough resources for the implementation of the software | | |
| Yes | 89 (67.9) | 35 (50.7) |
| Partly | 9 (6.9) | 7 (10.1) |
| No | 13 (9.9) | 18 (26.1) |
| I don’t know/ no answer | 20 (15.5) | 9 (13.0) |
| The organisational framework conditions supported the implementation of the software | | |
| Yes | 72 (55.0) | 28 (40.6) |
| Partly | 15 (11.5) | 11 (15.9) |
| No | 20 (15.3) | 19 (27.5) |
| I don’t know/ no answer | 24 (18.3) | 11 (15.9) |
| The management level supported the implementation of the software | | |
| Yes | 95 (72.5) | 41 (59.4) |
| Partly | 5 (3.8) | 7 (10.1) |
| No | 11 (8.4) | 10 (14.5) |
| I don’t know/ no answer | 20 (15.3) | 11 (15.9) |
| I value the support of the management level | | |
| Yes | 72 (55.0) | 39 (57.4) |
| Partly | 10 (7.6) | 9 (13.2) |
| No | 30 (22.9) | 7 (10.3) |
| I don’t know/ no answer | 19 (14.5) | 13 (19.1) |
| I think the implementation of the software is reasonable and justified | | |
| Yes | 53 (40.5) | 19 (27.5) |
| Partly | 27 (20.6) | 11 (15.9) |
| No | 45 (34.4) | 36 (52.2) |
| I don’t know/ no answer | 6 (4.6) | 3 (4.3) |
| Organisational framework conditions changed positive due to the implementation of the software (e.g. saving of time) | | |
| Yes | 13 (10.0) | 9 (13.0) |
| Partly | 14 (10.8) | 9 (13.0) |
| No | 94 (72.3) | 48 (69.6) |
| I don’t know/ no answer | 9 (6.9) | 3 (4.3) |

Table S6: Medical Context

|  | **Initial telephone contact point (116117)**  **n=130 (65.5%)** | **Joint Counter**  **n=69 (34.5%)** |
| --- | --- | --- |
| The initial assessments of the software are coincided with my own assessments or with those of an earlier software | | |
| Yes | 31 (23.7) | 29 (42.0) |
| Partly | 47 (35.9) | 28 (40.6) |
| No | 40 (30.5) | 7 (10.1) |
| I don’t know/ no answer | 13 (9.9) | 5 (7.2) |
| The initial assessments of the software are coincided with the assessment of an physician | | |
| Yes | 17 (13.0) | 21 (30.9) |
| Partly | 29 (21.1) | 24 (35.3) |
| No | 25 (19.1) | 6 (8.8) |
| I don’t know/ no answer | 60 (45.8) | 17 (25.0) |
| Patients with a high degree of urgency have been identified faster due to the software | | |
| Yes | 30 (22.9) | 10 (14.4) |
| Partly | 29 (21.1) | 26 (37.7) |
| No | 53 (40.5) | 28 (40.6) |
| I don’t know/ no answer | 19 (14.5) | 5 (7.2) |
| Patients with a low degree of urgency have been identified due to the software and were steered into the right point of care | | |
| Yes | 36 (27.5) | 12 (17.4) |
| Partly | 33(25.2) | 17 (24.6) |
| No | 38 (29.0) | 30 (43.5) |
| I don’t know/ no answer | 24 (18.3) | 10 (14.5) |
| The support by the software regarding patient counselling was useful | | |
| Yes | 33 (25.2) | 14 (20.3) |
| Partly | 38 (29.0) | 13 (18.8) |
| No | 52 (39.7) | 38 (55.1) |
| I don’t know/ no answer | 8 (6.1) | 4 (5.8) |
| I think the implementation of the software may increases risk of patient harm | | |
| Yes | 36 (27.7) | 17 (24.6) |
| Partly | 23 (17.7) | 9 (13.0) |
| No | 55 (42.3) | 22 (31.9) |
| I don’t know/ no answer | 16 (12.3) | 21 (30.4) |
| Patient satisfaction increased due to easier access to medical resources | | |
| Yes | 10 (7.6) | 8 (11.6) |
| Partly | 21 (16.0) | 5 (7.2) |
| No | 59 (45.0) | 35 (50.7) |
| I don’t know/ no answer | 41 (31.3) | 21 (30.4) |
| The use of the software reduced time needed per patient | | |
| Yes | 8 (6.1) | 6 (8.7) |
| Partly | 9 (6.9) | 6 (8.7) |
| No | 109 (83.2) | 50 (72.5) |
| I don’t know/ no answer | 5 (3.8) | 7 (10.1) |
| There was an contact person available in case of uncertainties regarding the medical assessment | | |
| Yes | 24 (18.3) | 17 (24.6) |
| Partly | 14 (10.7) | 14 (20.3) |
| No | 76 (58.0) | 29 (42.0) |
| I don’t know/ no answer | 17 (13.0) | 9 (13.0) |
